# Sporotrichosis refractory to conventional treatment: therapeutic success with potassium iodide^☆^^☆☆^

**DOI:** 10.1016/j.abd.2020.04.013

**Published:** 2021-01-31

**Authors:** Marcelo Rosandiski Lyra, Vanessa Sokoloski, Priscila Marques de Macedo, Anna Carolina Procópio de Azevedo

**Affiliations:** a Instituto Nacional de Infectologia Evandro Chagas, Fundação Oswaldo Cruz, Rio de Janeiro, RJ, Brazil; b Hospital Central Aristarcho Pessoa, Rio de Janeiro, RJ, Brazil

**Keywords:** Cryosurgery, Itraconazole, Potassium iodide, Sporothrix, Sporotrichosis

## Abstract

Sporotrichosis is a subcutaneous mycosis caused by dimorphic fungi of the genus *Sporothrix*. The authors report a case of fixed cutaneous sporotrichosis with therapeutic failure after 18 months of itraconazole and terbinafine associated with cryosurgery. The patient was cured after the introduction of saturated potassium iodide solution. *Sporothrix brasiliensis* was the identified species, presenting a susceptibility profile to itraconazole and terbinafine. This fact suggests that therapeutic failure is probably related to the host-fungus interaction rather than drug resistance. It is possible that the immunomodulatory action of the saturated potassium iodide solution may have played an important role in curing this patient.

Sporotrichosis is a subcutaneous mycosis caused by dimorphic fungi of the genus *Sporothrix*. It usually occurs through traumatic inoculation of the fungus in the skin or mucous membranes. The increase in cases in the state of Rio de Janeiro is related to the feline zoonotic epidemic that began in 1998; the main species involved was *Sporothrix brasiliensis*.^1^

The first choice for the treatment of lymphocutaneous sporotrichosis is itraconazole at a dose of 100 mg/day. Although robust experience attests to the safety and efficacy of itraconazole in the treatment of this disease, its metabolism depends on CYP3A4, which can lead to multiple drug interactions.^2^ Terbinafine is a safe and effective alternative when itraconazole is contraindicated.^3^

Saturated solution of potassium iodide (SSKI) was the first treatment for sporotrichosis through its immunological action, acting on the breakdown of granulomas, neutrophil chemotaxis, *Sporothrix* phagocytosis, and inhibition of biofilm in the yeast and filamentous phases.^4^, ^5^ It is also an alternative in cats refractory to itraconazole.^6^

Cryosurgery has been successfully used in 199 patients with slow or refractory responses to itraconazole/terbinafine.^7^

The authors report the case of a healthy 25-year-old woman with a left shoulder lesion of 75 days evolution (Fig. 1). She reported contact with a sick cat. The culture for fungi confirmed the diagnosis of sporotrichosis (Fig. 2). The clinical form was fixed cutaneous sporotrichosis.

**Figure 1:**
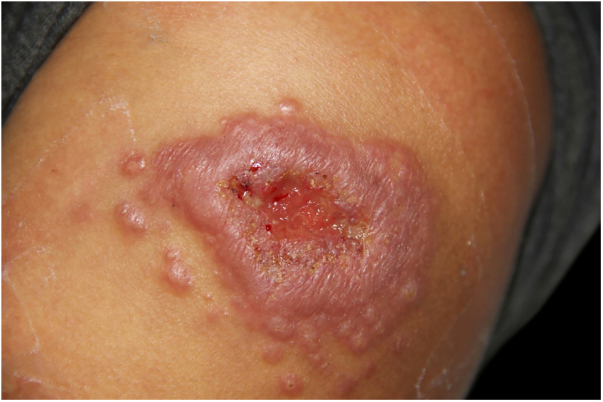
Infiltrated plaque with central ulceration surrounded by multiple satellite papules of 75 days evolution.

**Figure 2:**
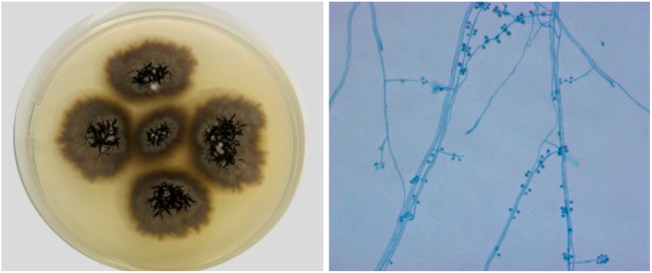
*Sporothrix brasiliensis* – left, colony macromorphology in the filamentous phase in potato dextrose agar (PDA), after 14 days of incubation at 30 °C. On the right, microscopy of the filamentous phase, stained by cotton blue with lactophenol (× 400).* The experiments of this work were carried out at the Mycology Laboratory of the National Institute of Infectious Diseases Evandro Chagas INI - Fiocruz/RJ.

She was treated with itraconazole 200 mg/day for 11 months without good response; terbinafine 250 mg/day was added to the regimen for another seven months with partial improvement. Simultaneously, she received 11 cryosurgery sessions at monthly intervals; however, the lesion remained infiltrated, with a central crust (Fig. 3).

**Figure 3:**
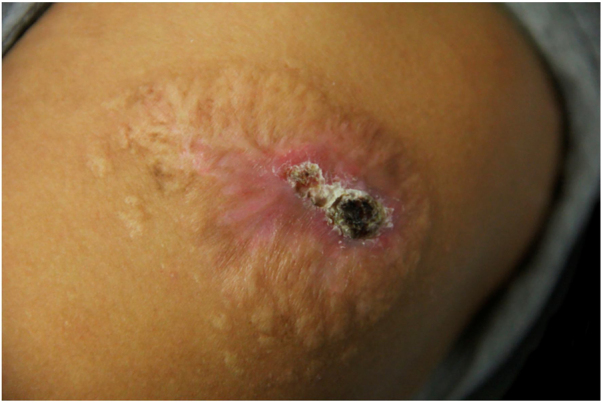
Infiltrated plaque with central sero-hematic crust after 18 months of treatment. Eleven months with itraconazole followed by seven months of terbinafine, combined with 11 sessions of cryosurgery at monthly intervals.

Due to the therapeutic failure, the authors chose to associate SSKI 1.42 g/mL (started at 0.6 g/day, with a progressive increase every three days up to the dose of 2.1 g/day). Two months after the start of SSKI, the lesion was completely healed (Fig. 4).

**Figure 4:**
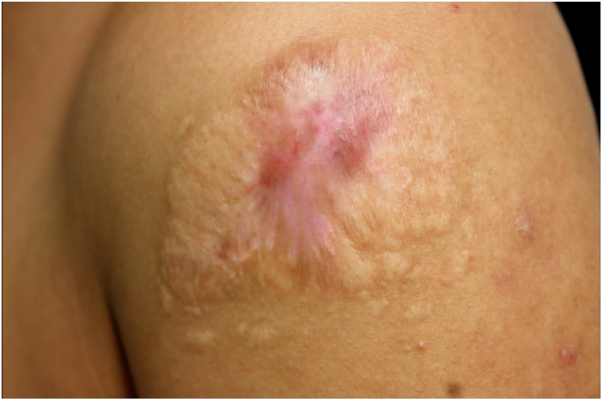
Lesion completely healed after 20 months of evolution and two months of treatment with saturated solution of potassium iodide.

The antifungal sensitivity profile of the clinical isolate molecularly identified as *S. brasiliensis*, performed using the CLSI M38-A2 method, showed the following minimum inhibitory concentrations (MIC): amphotericin B, 2.0 µg/mL; itraconazole, 1.0 µg/mL; terbinafine, 0.125 µg/mL. The experiments of this work were carried out at the Mycology Laboratory of the National Institute of Infectious Diseases Evandro Chagas INI - Fiocruz/RJ.

Cases of sporotrichosis refractory to conventional antifungal therapy are rare. Generally, patients with slow response evolve to cure after adjuvant cryosurgery.^7^

*S. brasiliensis* was shown to be more susceptible to antifungals than *S. schenckii*.^8^ Although rare, resistance to itraconazole/terbinafine has been described in the literature.^9^ Despite the favorable sensitivity profile, the present case had therapeutic failure. It is likely that therapeutic failure is related to the host-fungus interaction rather than drug resistance.

SSKI is no longer the first choice in the treatment of sporotrichosis due to several factors: lack of standardized commercial formulation, unknown mechanisms of action, metallic taste, and especially the advent of modern and safe antifungal drugs. Recently, alternative therapies with lower doses of SSKI have been proposed, with very promising results.^10^

The authors highlight SSKI as a safe and effective therapeutic alternative in the treatment of cutaneous sporotrichosis refractory to itraconazole/terbinafine, emphasizing that the immunomodulatory action of SSKI may have played an important role in curing this patient.

## Financial support

None declared.

## Authors’ contributions

Marcelo Rosandiski Lyra: Approval of the final version of the manuscript; design and planning of the study; drafting and editing of the manuscript; collection, analysis, and interpretation of data; effective participation in research orientation; intellectual participation in propaedeutic and/or therapeutic conduct of the studied cases; critical review of the literature; critical review of the manuscript.

Vanessa Sokoloski: Design and planning of the study; drafting and editing of the manuscript; critical review of the literature; critical review of the manuscript.

Priscila Marques de Macedo: Approval of the final version of the manuscript; drafting and editing of the manuscript; effective participation in research orientation; critical review of the literature; critical review of the manuscript.

Anna Carolina Procópio de Azevedo: Statistical analysis; drafting and editing of the manuscript; collection, analysis, and interpretation of data; critical review of the manuscript.

## Conflicts of interest

None declared.
